# Whis is the opinion of Italian psychiatrists regarding Telepsychiatry?

**DOI:** 10.1192/j.eurpsy.2022.445

**Published:** 2022-09-01

**Authors:** L. Orsolini, U. Volpe

**Affiliations:** 1 Polytechnic University of Marche, Department Of Clinical Neurosciences/dimsc, School Of Medicine, Unit Of Psychiatry, Ancona, Italy; 2 Unit of Clinical Psychiatric, Polytechnic University of Marche, Ancona, Italy, Department Of Neurosciences/dimsc, Ancona, Italy

**Keywords:** telepsychiatry, Covid-19, digitalization, digital psychiatry

## Abstract

**Introduction:**

Italy was the first European country to face up with COVID-19 pandemic, which posed challenges to National Health System (NHS), including the need to adapt mental health services/infrastructures and implement digitalization.

**Objectives:**

Despite telepsychiatry (ie., delivery of psychiatric care remotely through IT), is extensively used in non-European countries, only during the COVID-19 pandemic, became a convincing alternative to face-to-face modality for many psychiatrists in their clinical practice. Our aim was investigating Italian psychiatrists’ opinion about telepsychiatry.

**Methods:**

A questionnaire, disseminated during the third Italian phase, constituted by three sections (socio-demographic, opinions and personal experience about/with telepsychiatry) was build by adapting the 42-item questionnaire by Schubert (2019) and CAMH’s Client Experience Survey from the psychiatrist’s perspective.

**Results:**

90 questionnaires were collected from a sample of 54 women (60%) with an average age of 43(SD=11.4). Mostly were psychiatrists (85.6%) working in NHS (66.7%) with an average working years of 13.7(SD=11.5) and a previous experience in telepsychiatry (71.1%). Overall, participants do not believe that telepsychiatry is comparable with face-to-face modality. A significant positive opinion was reported among younger psychiatrists compared to those more experienced, regarding efficacy, feasibility and mental health access (p<0.05). No significant differences were reported in psychiatrists’ opinion, according to the level of telepsychiatry use in their clinical practice.

**Conclusions:**

Overall, sufficient digital skills and knowledge of technological tools are evident among younger psychiatrists who also appeared to be more prone to implement telepsychiatry in their clinical practice.

**Disclosure:**

No significant relationships.

